# The darker side of positive AI attitudes: Investigating associations with (problematic) social media use^☆^

**DOI:** 10.1016/j.abrep.2025.100613

**Published:** 2025-06-02

**Authors:** Christian Montag, Jon D. Elhai

**Affiliations:** a Centre for Cognitive and Brain Sciences, Institute of Collaborative Innovation, University of Macau, Macau SAR, PR China; b Department of Computer and Information Science, Faculty of Science and Technology, University of Macau, Macau SAR, PR China; c Department of Psychology, Faculty of Social Sciences, University of Macau, Macau SAR, PR China; d Department of Psychology, and Department of Neurosciences and Psychiatry, University of Toledo, Toledo, OH, USA

**Keywords:** Artificial Intelligence, Attitudes, Problematic Social Media Use, Social Media Addiction

## Abstract

In societies around the world, the impact of artificial intelligence (AI) is being fiercely discussed. It is difficult to grasp AI’s influence, because AI represents a general-purpose technology, which can be applied in different settings. One product in which AI plays a pivotal role is social media. In this context, for instance, AI is used to provide people with personalized newsfeeds to prolong time spent online, which might result in addictive-like behavior. Many factors such as sociodemographic variables, history of psychopathology and personality traits have been revealed as risk factors for developing problematic social media use patterns. Yet, to our knowledge attitudes toward AI have not been examined in association with problematic social media use. In a sample of n = 956 social media users, we observed that positive AI attitudes were linked to overuse of social media as assessed with an addiction framework. The effect size of this association was stronger for males than females. Further we observed that this association was mediated by time spent on social media. The present study shows that positive AI attitudes – although well-known to be positive regarding embracing new technologies – might come with risks for developing addictive patterns of technology use, such as social media.

## Highlights

• Associations between AI attitudes and social media use are studied.
• Positive AI attitudes link to more problematic social media use.
• The present study shows that positive AI attitudes might come with costs for users.

## 1 Introduction

At the moment of writing, more than five billion people use social media (Statista, 2022). Social media represents a product which heavily relies on artificial intelligence (AI), for instance to present users with a personalized newsfeed such as on Facebook or to recommend videos to users on platforms such as YouTube (Guha, 2021). Social media platforms have been designed in such a way to prolong users’ time spent online (Montag and Elhai, 2023, Sindermann et al., 2022). Consequently, people increasingly leave more digital footprints on the platforms, which can be exploited to gain important insights into user characteristics to target users with personalized ads (Matz et al., 2017, Zarouali et al., 2020). The big data business model has been critiqued from various angles such as loss of privacy and worsening well-being (Montag & Hegelich, 2020). In the context of addictive behaviors, it has been debated how the design of social media platforms might be responsible for development of addictive-like social media use (Flayelle et al., 2023, Montag et al., 2019, Montag et al., 2022). In this regard, AI plays a pivotal role, because without this technology it would be impossible to personalize social media (Salma et al., 2024), as detailed above.

In the past, several theoretical models have been developed to predict who may develop problematic social media use patterns (studied within an addiction framework). For instance, the prominent I-PACE model (Brand et al., 2016, Brand et al., 2019), involving the Interaction of Person, Affect, Cognition and Execution variables, conceptualizes that among the Person/dispositional variables, specific sociodemographic variables such as age, history of psychopathology, genetics or personality might predict problematic social media use (PSMU). Additionally, other risk factors such as affective and cognitive responses, and Internet-related cognitive biases, play a role in influencing development or maintenance of PSMU. In this context we propose that AI attitudes might also play an important role. We believe that AI attitudes might belong to the C-variable within the I-PACE model, because they could represent a cognitive response to the rise of this new technology.

Classic theories such as the Theory of Planned Behavior (TPB) (Ajzen, 1991) or Technology Acceptance Model (TAM) (Venkatesh & Davis, 2000) put forward the proposal that a positive attitude toward technology is an important prerequisite to use a technology. These theories and the proposals have been recently transferred to AI use (Montag & Ali, 2025b). Positive AI attitudes therefore might be seen as something promising, because they might help people embrace new technologies, making them more productive in everyday life tasks. On the other hand, such positive AI attitudes might result in overreliance on AI technologies or overuse tendencies (see recent works on overreliance on AI; Buçinca et al., 2021, Klingbeil et al., 2024). As social media represents a product strongly relying on AI, we were interested in understanding if positive AI attitudes link to PSMU severity. For reasons of completeness, we also investigated negative AI attitudes with PSMU, where inverse associations were expected. A further reflection on the hypotheses is necessary: Social media represents a product where AI is built-in, which users may not necessarily be aware of. Hence the question arises – also in light of TPB and TAM – whether users with more positive AI attitudes actively choose social media use (or develop excessive social media use) due to its AI character or if this happens due to being in general more tech-savvy. This is something we will reflect on deeper in the discussion.

## 2 Methods

### 2.1 On the investigated sample

The sample here was already described in Montag et al. (2023) and analyzed regarding associations between PSMU, meaning in life and fear of missing out. The larger dataset also included additional variables, which allowed investigation of other research questions such as trusting AI (Montag et al., 2024) and an investigation of links between Shinrin-Yoku and life satisfaction (Montag, 2024). These projects were all preregistered. For the present work, we included PSMU as investigated in Montag et al. (2023) and Attitudes toward AI (ATAI) as investigated by Montag, Becker & Li (Montag et al., 2024). Please note that the papers differ slightly regarding the final sample sizes due to slightly different data cleaning steps from adding measures. For the present paper please see the data cleaning steps in the next section. The present paper investigates, for the first time, associations between attitudes toward AI and PSMU, which has not been done in the above mentioned papers in this section.

### 2.2 Data cleaning

An initial sample of 1151 participants was recruited via Bilendi GmbH (a company supporting scientists in conducting online surveys). The company was asked to recruit a population sample with about the same number of male and female participants with a large age-range (to not rely on typically available student samples). After excluding participants who did not answer the survey without interruptions, participants reporting a third gender (unfortunately underrepresented), not providing full consent, failing an attention item, or not being competent in the German language, we ended up with 1082 participants. Please note that we refrained from doing careless responding analysis on measures not relevant to the present paper. Beyond the paper by Montag et al. (2023) we also investigated time spent on social media. Those who reported spending more than 16 h a day on social media (personal and work combined) were excluded as outliers. We further checked for missing data or computation errors, which led to a final sample of n = 1048 participants (523 males, 525 females; mean-age: 45,0, SD = 14,4). Education level was as follows (for precision, German words are used): without school = 0.2 %, Volks-/Hauptschulabschluss (Primary/Secondary School Certificate): 7.8 %, Mittlere Reife (Intermediate School Certificate): 28.6 %, Fachabitur (Advanced Technical College Entrance Qualification): 7.3 %, Abitur (General University Entrance Qualification): 20.2 %, Fachhochschulabschluss (Advanced Technical College Degree): 12.5 %, Hochschulabschluss (university degree): 23.4 %. The education degrees are presented in ascending order. Please note that the final sample of social media users differs from our earlier work on PSMU (stemming from the same data set; Montag et al., 2023): 955 vs. 956 participants given a slightly different data cleaning approach.

The study was approved by the ethics committee at Ulm University, Ulm, Germany.

### 2.3 Questionnaires

The German and English versions of the ATAI each consist of five items, here answered via a five-point Likert scale ranging from “1 = strongly disagree” to “5 = strongly agree” (Sindermann et al., 2021). Two items form the acceptance of AI scale (α = 0.77, ω = 0.77), and three items form the fear of AI scale (α = 0.77, ω = 0.78). The two acceptance items are: “I trust artificial intelligence” and “Artificial intelligence will benefit humankind”. The three fear items are: “I fear artificial intelligence”, “Artificial intelligence will destroy humankind” and “Artificial intelligence will cause many job losses”. Higher scores indicate greater acceptance of AI or greater fear of AI, respectively.

In addition, two single items were investigated, allowing us to obtain insights into global AI attitudes. These items included “I have a positive attitude toward AI” and “I have a negative attitude toward AI.” These items are also answered via a five-point Likert scale ranging from “1 = strongly disagree” to “5 = strongly agree”. The items have been validated recently (Montag et al., 2025, Montag and Ali, 2025a, Naiseh et al., 2025).

Social media use and PSMU were assessed as follows: First, participants were asked if they use social media (original wording translated from the German item: “I use social media. This includes platforms such as Facebook, Instagram, TikTok, YouTube, LinkedIn, Snapchat, as well as messaging apps like WhatsApp, Signal, or Telegram.”; yes = 956 / no = 92). If reporting use of social media, they were subsequently asked about the use for personal and work purposes in minutes per day (average estimates). Finally, they completed the Social Networking Sites – Addiction Test (SNS-AT) consisting of six items answered on a five-point Likert scale ranging from “1 = strongly disagree” to “5 = strongly agree” (Montag et al., 2023). The SNS-AT assesses individual differences in PSMU tendencies, with higher scores indicating greater tendencies. Internal consistency for the SNS-AT was as follows: α = 0.89, ω = 0.89.

### 2.4 Statistical Analyses

Data cleaning was conducted with SPSS 30.0.0.0. The next statistical analyses were computed with the Jamovi package 2.3.28.0. Descriptive statistics were produced first, which were followed by t-tests providing insights into associations between social media use (yes/no) and attitudes toward AI. Also t-tests/Mann-Whitney U tests were computed to assess gender effects based on the smaller sample of social media users. For this investigation we used Julius.ai to find a (near) perfect match for the n = 92 participants reporting to not use social media regarding the variables of gender, age and education (in the following order of importance in the matching process). The samples are comparable regarding gender (each sample including 54 males and 38 females), near identical regarding age (non-users: M = 53,05, SD = 12,56 vs. users: M = 53,04, SD = 12,55; t_(182)_ = 0,006, p = 0.995) and education (Chi^2^ = 4.60, df = 5, p = 0.47). This strategy was chosen in order to avoid testing a small sample such as 92 vs. 956 participants.

Further, correlations between all variables of interest are presented in a next step for the male and female subsamples of social media users. Here we focused on Spearman correlations due to the skewed distribution of several variables. Finally, within this slightly smaller social media use sample (males plus females), a mediation model is presented (using Mplus 8.11 software) investigating associations between positive AI attitudes (predictor variable) and PSMU (outcome variable) with time spent on social media (personal + work time) being a mediator. We used maximum likelihood estimation for mediation, using the Delta method to compute cross-products of direct path coefficients with 1000 non-parametric bootstrapped replications; we present standardized (STDYX) coefficients. Predictor and outcome terms do not imply causality here.

## 3 Results

First, we present descriptive statistics for AI attitudes, social media use and problematic use in Table 1. The social media variables used the smaller sample of social media users. All descriptive statistics are also presented for the male and female subsamples in Table 2 (given several gender differences being backed up by S-Table 1 via t-tests and Mann-Whitney U tests in the supplementary material; some variables had skewed distributions so we provide insights using both parametric and non-parametric tests).

**Table 1:** Descriptive statistics of the complete sample.

	**N**	**Missing**	**Mean**	**Median**	**SD**	**Minimum**	**Maximum**
Accepting AI (ATAI + )	1048	0	6.19	6.00	1.755	2	10
Fear of AI (ATAI −)	1048	0	8.20	8.00	2.690	3	15
Single item: AI +	1048	0	3.14	3.00	0.996	1	5
Single item: AI −	1048	0	2.76	3.00	1.120	1	5
SNS − AT	956	92	13.04	12.00	5.613	6.00	30.0
TSSM	956	92	170.59	120.00	169.944	0.00	920.0

ATAI: Attitudes for AI scale with acceptance (ATAI + ) and fear (ATAI −) subscales; Single item framework: AI attitudes positive (AI + ) and negative (AI −) with one item each; SNS-AT: Social Networking Sites-Addiction Test; TSSM: Time spent on social media (aggregate of personal and business time per day in minutes).

**Table 2:** Descriptives statistics for male and female social media users under investigation.

	**Gender**	**N**	**Missing**	**Mean**	**Median**	**SD**	**Minimum**	**Maximum**
Accepting AI (ATAI + )	Male	469	0	6.52	6	1.822	2	10
	Female	487	0	6.03	6	1.612	2	10
Fear of AI (ATAI −)	Male	469	0	7.73	8	2.617	3	15
	Female	487	0	8.55	9	2.701	3	15
Single item (AI + )	Male	469	0	3.35	3	0.992	1	5
	Female	487	0	3.04	3	0.959	1	5
Single item (AI −)	Male	469	0	2.62	3	1.140	1	5
	Female	487	0	2.82	3	1.086	1	5
SNS-AT	Male	469	0	13.59	13.0	6.067	6.00	30.0
	Female	487	0	12.50	12.0	5.088	6.00	30.0
TSSM	Male	469	0	165.73	90.0	174.202	2.00	920.0
	Female	487	0	175.26	120.0	165.786	0.00	900.0
Age	Male	469	0	45.49	44.0	13.519	19.00	83.0
	Female	487	0	43.00	42.0	14.886	18.00	73.0

ATAI: Attitudes for AI scale with acceptance (ATAI + ) and fear (ATAI −) subscales; Single item framework: AI attitudes positive (AI + ) and negative (AI −) with one item each; SNS-AT: Social Networking Sites-Addiction Test; TSSM: Time spent on social media (aggregate of personal and business time per day in minutes).

Second, we investigated if using social media (yes/no) was associated with differences in AI attitudes. We investigated this question in social media and non-social-media users being matched regarding sociodemographics as reported in the statistical analyses section. Among others it could be observed that users of social media had greater positive AI attitudes than those not using social media (ATAI +: t_(182)_ = 3.34, p < 0.001, M = 6,10 (SD = 1,52) vs. M = 5,29 (SD = 1,74), Cohen’s d = 0.492; single item AI +: t_(182)_ = 3.96, p < 0.001, M = 3,20 (SD = 0,91) vs. M = 2,65 (SD = 0,95), Cohen’s d = 0.585).

Among users of social media, we further investigated how AI attitudes would be linked to time spent on social media and PSMU tendencies. Given the effects of gender on some of the variables, we present correlation patterns for males and females separately (Table 3, Table 4). For both genders we observed that the acceptance of AI scale and single item positive AI attitude measure were positively linked to both time-spent on social media and PSMU scores. Associations between positive AI attitudes and PSMU were in the small to moderate range for males and in the very mild area for females. The negative AI attitudes scales showed no robust associations with social media use or PSMU (but see a small positive association in the male subsample for the ATAI −). Please note that we report here correlations controlled for age. The correlation tables not controlled for age can be found in the supplementary material (see S-Table 2 and S-Table 3).

**Table 3:** Spearman correlations for male social media users (controlling for age).

		**ATAI+**	**ATAI-**	**Single item: AI +**	**Single item: AI −**	**TSSM**	**SNS-AT**
Acceptance of AI							
(ATAI + )	Spearman's rho	—					
	p-value	—					
Fearing AI							
(ATAI −)	Spearman's rho	−0.502	—				
	p-value	<.001	—				
Single item: AI +	Spearman's rho	0.704	−0.480	—			
	p-value	<.001	<.001	—			
Single item: AI −	Spearman's rho	−0.499	0.614	−0.574	—		
	p-value	<.001	<.001	<.001	—		
TSSM	Spearman's rho	0.135	−0.005	0.136	−0.045	—	
	p-value	0.003	0.913	0.003	0.330	—	
SNS-AT	Spearman's rho	0.266	0.107	0.270	−0.009	0.351	—
	p-value	<.001	0.020	<.001	0.848	<.001	—

Note. controlling for 'age'.

ATAI: Attitudes for AI scale with acceptance (ATAI + ) and fear (ATAI −) subscales; Single item framework: AI attitudes positive (AI + ) and negative (AI −) with one item each; SNS-AT: Social Networking Sites-Addiction Test; TSSM: Time spent on social media (aggregate of personal and business time per day in minutes).

**Table 4:** Spearman correlations for female social media users (controlling for age).

		**ATAI +**	**ATAI −**	**Single item: AI+**	**Single item: AI −**	**TSSM**	**SNS-AT**
Acceptance of AI (ATAI + )	Spearman's rho	—					
	p-value	—					
Fear of AI (ATAI −)	Spearman's rho	−0.531	—				
	p-value	<.001	—				
Single item: AI +	Spearman's rho	0.725	−0.619	—			
	p-value	<.001	<.001	—			
Single item: AI −	Spearman's rho	−0.641	0.691	−0.786	—		
	p-value	<.001	<.001	<.001	—		
TSSM	Spearman's rho	0.089	−0.071	0.104	−0.128	—	
	p-value	0.049	0.116	0.022	0.005	—	
SNS-AT	Spearman's rho	0.118	0.023	0.105	−0.024	0.294	—
	p-value	0.009	0.610	0.021	0.590	<.001	—

Note. controlling for 'age'.

ATAI: Attitudes for AI scale with acceptance (ATAI + ) and fear (ATAI −) subscales; Single item framework: AI attitudes positive (AI + ) and negative (AI −) with one item each; SNS-AT: Social Networking Sites-Addiction Test; TSSM: Time spent on social media (aggregate of personal and business time per day in minutes).

Basing on the correlational analysis, we prepared an example of a mediation model suggesting that the association between more positive AI attitudes (ATAI +: acceptance of AI) and greater PSMU tendencies is mediated by time spent on social media (combined personal and business time). This model was based on the idea that AI attitudes (positive) could result in prolonged use time on social media which might consequently result in addictive social media tendencies. Also, alternative sequences of variables are possible of course, given the cross-sectional nature of the dataset. Next, we conducted a slight revision of this model, adding paths from age and gender (as covariates) to PSMU, finding that even with age and gender variables, the indirect/mediation effect was still significant (see Table 5 and Table 6).

**Table 5:** Standardized Mediation Estimates (adding age and gender covariates).

	**95 % Confidence Interval**						
**Effect**	**Label**	**Estimate**	**SE**	**Lower**	**Upper**	**Z**	**p**
Indirect	a × b	0.031	0.010	0.015	0.048	3.144	0.002

Note: Table 6 outlines the lettered labels in terms of how the products of path coefficients were computed.

**Table 6:** Standardized Path Estimates (adding age and gender covariates).

	**95 % Confidence Interval**									
			**Label**	**Estimate**	**SE**	**Lower**	**Upper**	**Z**	**p**	
ATAI +	→	TSSM	a	0.121	0.035	0.063	0.178	3.443	0.001	
TSSM	→	SNS-AT	b	0.261	0.029	0.212	0.309	8.82	0<.001	
ATAI +	→	SNS-AT	c	0.205	0.031	0.153	0.256	6.511	0<.001	
Age	→	SNS-AT	d	−0.336	0.027	−0.381	−0.291	−12.264	0<.001	
Gender	→	SNS-AT	e	−0.215	0.055	−0.306	−0.123	−3.868	0<.001	

ATAI +: Acceptance of AI; TSSM: Time spent on social media (aggregate of personal and business time per day in minutes).

## 4 Discussion

The present study investigated associations between global AI attitudes and both social media use and overuse. We discovered that positive AI attitudes were linked with using social media (yes vs. no) and to higher PSMU scores. These findings could provide evidence that more positive AI attitudes might be a risk factor for developing greater PSMU levels (but causality cannot be inferred from the present data). Social media itself relies heavily on AI technology explaining why this link could be established. Interestingly, negative AI attitudes showed less robust associations with social media use patterns. This was not expected, but is in line with prior observations that acceptance of AI might be more important to understand why people embrace AI technology in contrast to the role of fearing AI (Sindermann et al., 2021).

The findings observed here therefore not only underline that positive AI attitudes are associated with PSMU, but also that it is meaningful to distinguish between positive and negative AI attitudes (hence not thinking of the constructs as one dimension with two poles). This finding needs to be tested in the future by applying further AI attitude inventories and also focusing more on the role of negative AI attitudes. Here we would also not be surprised to see positive associations with PSMU (in contrast to our initial hypothesis), perhaps due to the link of negative emotions being part of both constructs (and perhaps those fearing AI might want to escape their negative emotions by excessively checking social media). But this is something we have only seen in the male subsample (small effect size of about .10). Other inventories to be tested are for instance the ATTARI-12, which has been put forward as a one-dimensional attitude towards AI scale (Stein, Messingschlager, Gnambs, Hutmacher, & Appel, 2024), which could also be investigated regarding potential links with PSMU (and then contrasted with the present findings). For a further discussion on AI attitude measures, see a recent chapter providing a good overview (Schepman & Rodway, 2025). Further research options of course would be the longitudinal investigation of AI attitudes and PSMU to shed light on the question of whether positive AI attitudes indeed might be a risk factor for developing PSMU.

Another point to reflect on has been mentioned shortly at the end of the introduction. Although social media relies heavily on artificial intelligence technology, people not necessarily might be aware of it. When interacting with a large language model such as ChatGPT, a person actively starts to use an AI agent to obtain information. On a social media platform, you might simply log on and browse and indirectly rely on what the AI recommends to you. Hence, it is unclear if PSMU is indeed linked to positive AI attitudes or perhaps a construct such as general tech-savviness overlapping with such positive attitudes. Therefore, also the question arises, if a positive attitude toward AI is not only linked to PSMU, but also to other problematic online behaviors. Hence, the question can be posed if having a positive AI attitude and being more tech-savvy makes a person more vulnerable to overuse technologies in general (e.g. developing generalized problematic Internet use behaviors; Davis, 2001). But it could also be the case that positive AI attitudes map more onto specific problematic online behaviors, in particular if certain platforms in the area of gaming/gambling/shopping, etc. would rely more on AI-technology than others. As briefly mentioned, awareness of the degree of AI being used in these different online environments might have an influence on the association strength between positive AI attitudes and a certain problematic online behavior, for instance that people are more willing to use a videogame, which is AI empowered, when they really have a positive view on AI. This said, we might also see bidirectional effects here: more positive attitudes towards AI might result in more (excessive) AI technology use and the positive experience made with the AI technology might further shape the attitudes towards AI. AI being built into products such as videogames (Tang et al., 2020) could lead to more immersion and longer play time, hence shaping user behavior of a platform. How AI in its many forms will be able to shape human behavior toward more compulsive or impulsive use of technology (as an example) is up to further discussion. With the present data, we unfortunately cannot further shed light on several of the here mentioned issues, but want to point to such an interesting future research endeavor.

We further mention that the association between positive AI attitudes and PSMU severity was stronger in males than females. The separate analysis for males and females is important, because we detected gender effects, with males having more positive AI attitudes than females (see supplementary material). If positive AI attitudes will be carved out in the future as a risk factor for PSMU, it could be the case that males – being in particular open to new technologies – might more easily develop PSMU tendencies. In any case, it will be important to further take into account gender in the near future in this line of research.

Although the present work cannot prove that positive AI attitudes are causally linked to PSMU, the question arises, if use of artificial intelligence in digital products might alter products in such a way that they are getting more immersive (Montag, Yang, et al., 2025) and that certain user groups (here characterized by more positive AI attitudes) might in particular be drawn into longer use or show more addictive-like use. The latter also makes an interesting research question, namely if AI being built into a product such as social media simply results in prolonged use or pathological use of a product. Insights from such research for sure will also raise ethical concerns. Past research suggests that certain personality tendencies indeed might be more responsive to platform design (whereas AI can be also seen as such a feature; Sindermann, Montag, & Elhai, 2022). In this context, a controversial question arises when thinking of AI being used in a very critical area of social media, namely personalization of content or use of recommendation engines (Guha, 2021, Salma et al., 2024). AI can be seen as very helpful, because it selects for the user from millions of content pieces with a high chance of choosing those which might be most interesting (derived from the study of digital footprints left behind). This reduces cognitive burden on the user’s side, but might also result in filter bubbles (Pariser, 2011). For the users this kind of personalization might also dramatically increase engagement with the platform and online time – fostering PSMU. Therefore, there is a fine line between using AI for reducing cognitive burden and providing a meaningful service and on the other hand escalating online time, which is at the heart of the data business model (Montag & Elhai, 2023).

Although we did not study ChatGPT addiction in the present work, we want to reflect on this topic in the context of our studied AI attitudes (which obviously is a very different construct). Recent discussion around the concept of ChatGPT addiction arose (Lin and Chien, 2024, Yankouskaya et al., 2025), when a number of researchers started to investigate addictive use of ChatGPT. It is important to note, that so far the literature does not make a compelling case for use of the addiction term in this context and the danger of over-pathologizing behavior is a valid concern (Ciudad-Fernández et al., 2025). We believe that at the moment over-reliance on AI and the forming of parasocial relationships with LLMs such as character.ai, where the user bonds with a fictional (or celebrity) character, might be more relevant issues to be discussed around the intense use of LLMs (Montag et al., 2025). But an understanding of how addictive behaviors towards LLMs (again we are hesitant to use the term ChatGPT Addiction, etc.) might link to other established addictive behaviors can be interesting (and here also the link to AI attitudes could be studied again). We explicitly mention that this is not the research question of the present paper, because here we investigated the non-clinical construct of positive attitudes toward AI, whereas positive views on technology in the literature are well-established to help people embrace technology (with benevolent technology, this is something good). The controversy from the present data arises by also highlighting potential negative effects of such a positive view.

The present work comes with several limitations. The cross-sectional character prevents us from confidently inferring causality between the variables. Second, the work relies on self-report methodology including the usual problems such as lack of introspection, response bias, etc. Third, we used global measures of AI attitudes, whereas it might be more meaningful to investigate AI attitudes in a finer grained manner, such as attitudes toward AI within social media products (and here also asking study participants about their awareness of AI being built into social media). Recent research suggests that global AI attitudes have predictive power for use in specific areas though (Montag and Ali, 2025a, Sindermann et al., 2021). Fourth, the present work showed that age plays a role to understand associations between AI attitudes and PSMU and the present sample was relatively old (referring to the mean of the sample). As younger people might be more tech-interested or more open to use AI technology, this needs to be further investigated. Finally, future research should also consider investigating which social media AI elements people interact with, and to what extent.

In conclusion, the present work shows that AI attitudes might be relevant to understanding PSMU. However, the present research is exploratory and should only be seen as a first approach to understanding this area. Replication of the present findings will be needed.

## CRediT authorship contribution statement

**Christian Montag:** Writing – original draft, Methodology, Investigation, Formal analysis, Data curation, Conceptualization. **Jon D. Elhai:** Writing – review & editing, Methodology, Formal analysis.

## Declaration of competing interest

The authors declare that they have no known competing financial interests or personal relationships that could have appeared to influence the work reported in this paper.

## Data Availability

Data will be made available on request.
