# Supplementary material for: The darker side of positive AI attitudes: Investigating associations with (problematic) social media use
Source: Addict Behav Rep. 2025 Jun 2;22:100613. doi: 10.1016/j.abrep.2025.100613 (PMC12167016; doi:10.1016/j.abrep.2025.100613)
Supplement: Supplementary Data 1 [file mmc1.docx]

**Supplementary material**

| S-Table 1: Testing gender effects | | | | | | | | | | | | | | |
| --- | --- | --- | --- | --- | --- | --- | --- | --- | --- | --- | --- | --- | --- | --- |
|  | |  | | **Statistic** | | **df** | | **p** | |  | | **Effect Size** | | |
| Acceptance of AI (ATAI +) |  | Student's t |  | 4.328 | ᵃ | 954 |  | < .001 |  | Cohen's d |  | 0.2800 | |  |
|  |  | Mann-Whitney U |  | 97618 |  |  | | < .001 |  | Rank biserial correlation |  | 0.1452 | |  |
| Fear of AI (ATAI -) |  | Student's t |  | -4.771 |  | 954 |  | < .001 |  | Cohen's d |  | -0.3087 | |  |
|  |  | Mann-Whitney U |  | 94155 |  |  | | < .001 |  | Rank biserial correlation |  | 0.1755 | |  |
| Single item: AI + |  | Student's t |  | 4.988 | ᵃ | 954 |  | < .001 |  | Cohen's d |  | 0.3227 | |  |
|  |  | Mann-Whitney U |  | 94724 |  |  | | < .001 |  | Rank biserial correlation |  | 0.1706 | |  |
| Single item: AI + |  | Student's t |  | -2.763 | ᵃ | 954 |  | 0.006 |  | Cohen's d |  | -0.1787 | |  |
|  |  | Mann-Whitney U |  | 102266 |  |  | | 0.004 |  | Rank biserial correlation |  | 0.1045 | |  |
| TSSM |  | Student's t |  | -0.867 |  | 954 |  | 0.386 |  | Cohen's d |  | -0.0561 | |  |
|  |  | Mann-Whitney U |  | 106001 |  |  | | 0.054 |  | Rank biserial correlation |  | 0.0718 | |  |
| SNS-AT |  | Student's t |  | 3.013 | ᵃ | 954 |  | 0.003 |  | Cohen's d |  | 0.1949 | |  |
|  |  | Mann-Whitney U |  | 104311 |  |  | | 0.020 |  | Rank biserial correlation |  | 0.0866 | |  |
| Age |  | Student's t |  | 2.700 | ᵃ | 954 |  | 0.007 |  | Cohen's d |  | 0.1747 | |  |
|  |  | Mann-Whitney U |  | 102562 |  |  | | 0.006 |  | Rank biserial correlation |  | 0.1019 | |  |
| Note. Hₐ μ _männlich_ ≠ μ _weiblich_ | | | | | | | | | | | | | | |
| ᵃ Levene's test is significant (p < .05), suggesting a violation of the assumption of equal variances | | | | | | | | | | | | | | |
| ATAI: Attitudes for AI scale with acceptance (ATAI+) and fear (ATAI-) subscales; Single item framework: AI attitudes positive (AI+) and negative (AI-) with one item each; SNS-AT: Social Networking Sites-Addiction Test; TSSM: Time spent on social media (aggregate of personal and business time per day in minutes). | | | | | | | | | | | | | |  |
|  | | | | | | | | | | | | | | |

| S-Table 2: Correlation matrix of the male sample using social media (n = 469 males) | | | | | | | | | | | | | | | | | |
| --- | --- | --- | --- | --- | --- | --- | --- | --- | --- | --- | --- | --- | --- | --- | --- | --- | --- |
|  | |  | | **ATAI+** | | **ATAI-** | | **Single item: AI+** | | **Single item: AI-** | | **TSSM** | | **SNS-AT** | | **Age** | |
| ATAI+ |  | Spearman's rho |  | — |  |  |  |  |  |  |  |  |  |  |  |  |  |
|  |  | p-value |  | — |  |  |  |  |  |  |  |  |  |  |  |  |  |
| ATAI- |  | Spearman's rho |  | -0.493 |  | — |  |  |  |  |  |  |  |  |  |  |  |
|  |  | p-value |  | < .001 |  | — |  |  |  |  |  |  |  |  |  |  |  |
| Single item: AI+ |  | Spearman's rho |  | 0.721 |  | -0.474 |  | — |  |  |  |  |  |  |  |  |  |
|  |  | p-value |  | < .001 |  | < .001 |  | — |  |  |  |  |  |  |  |  |  |
| Single item: AI- |  | Spearman's rho |  | -0.490 |  | 0.615 |  | -0.566 |  | — |  |  |  |  |  |  |  |
|  |  | p-value |  | < .001 |  | < .001 |  | < .001 |  | — |  |  |  |  |  |  |  |
| TSSM |  | Spearman's rho |  | 0.204 |  | -0.014 |  | 0.198 |  | -0.051 |  | — |  |  |  |  |  |
|  |  | p-value |  | < .001 |  | 0.766 |  | < .001 |  | 0.266 |  | — |  |  |  |  |  |
| SNS-AT |  | Spearman's rho |  | 0.338 |  | 0.086 |  | 0.332 |  | -0.019 |  | 0.434 |  | — |  |  |  |
|  |  | p-value |  | < .001 |  | 0.062 |  | < .001 |  | 0.678 |  | < .001 |  | — |  |  |  |
| Age |  | Spearman's rho |  | -0.252 |  | 0.028 |  | -0.226 |  | 0.027 |  | -0.319 |  | -0.408 |  | — |  |
|  |  | p-value |  | < .001 |  | 0.543 |  | < .001 |  | 0.556 |  | < .001 |  | < .001 |  | — |  |
| ATAI: Attitudes for AI scale with acceptance (ATAI+) and fear (ATAI-) subscales; Single item framework: AI attitudes positive (AI+) and negative (AI-) with one item each; SNS-AT: Social Networking Sites-Addiction Test; TSSM: Time spent on social media (aggregate of personal and business time per day in minutes). | | | | | | | | | | | | | | | | | |

| S-Table 3: Correlation matrix of the female sample using social media (n = 487 females) | | | | | | | | | | | | | | | | | |
| --- | --- | --- | --- | --- | --- | --- | --- | --- | --- | --- | --- | --- | --- | --- | --- | --- | --- |
|  | |  | | **ATAI +** | | **ATAI -** | | **Single item: AI+** | | **Single item: AI-** | | **TSSM** | | **SNS-AT** | | **Age** | |
| ATAI+ |  | Spearman's rho |  | — |  |  |  |  |  |  |  |  |  |  |  |  |  |
|  |  | p-value |  | — |  |  |  |  |  |  |  |  |  |  |  |  |  |
| ATAI- |  | Spearman's rho |  | -0.526 |  | — |  |  |  |  |  |  |  |  |  |  |  |
|  |  | p-value |  | < .001 |  | — |  |  |  |  |  |  |  |  |  |  |  |
| Single item: AI+ |  | Spearman's rho |  | 0.720 |  | -0.620 |  | — |  |  |  |  |  |  |  |  |  |
|  |  | p-value |  | < .001 |  | < .001 |  | — |  |  |  |  |  |  |  |  |  |
| Single item: AI- |  | Spearman's rho |  | -0.632 |  | 0.692 |  | -0.786 |  | — |  |  |  |  |  |  |  |
|  |  | p-value |  | < .001 |  | < .001 |  | < .001 |  | — |  |  |  |  |  |  |  |
| TSSM |  | Spearman's rho |  | 0.111 |  | -0.050 |  | 0.085 |  | -0.090 |  | — |  |  |  |  |  |
|  |  | p-value |  | 0.015 |  | 0.270 |  | 0.059 |  | 0.046 |  | — |  |  |  |  |  |
| SNS-AT |  | Spearman's rho |  | 0.139 |  | 0.040 |  | 0.081 |  | 0.011 |  | 0.412 |  | — |  |  |  |
|  |  | p-value |  | 0.002 |  | 0.382 |  | 0.074 |  | 0.809 |  | < .001 |  | — |  |  |  |
| Age |  | Spearman's rho |  | -0.075 |  | -0.042 |  | 0.029 |  | -0.074 |  | -0.376 |  | -0.446 |  | — |  |
|  |  | p-value |  | 0.100 |  | 0.350 |  | 0.520 |  | 0.105 |  | < .001 |  | < .001 |  | — |  |
| ATAI: Attitudes for AI scale with acceptance (ATAI+) and fear (ATAI-) subscales; Single item framework: AI attitudes positive (AI+) and negative (AI-) with one item each; SNS-AT: Social Networking Sites-Addiction Test; TSSM: Time spent on social media (aggregate of personal and business time per day in minutes). | | | | | | | | | | | | | | | | | |
